# Ozone eliminates novel coronavirus Sars-CoV-2 in mucosal samples

**DOI:** 10.1016/j.nmni.2021.100927

**Published:** 2021-07-24

**Authors:** F. Sallustio, G. Cardinale, S. Voccola, A. Picerno, P. Porcaro, L. Gesualdo

**Affiliations:** 1) Department of Interdisciplinary Medicine, University of Bari “Aldo Moro”, Bari, Italy; 2) Consorzio Sannio Tech, Apollosa, Benevento, Italy; 3) Genus Biotech, Apollosa, Benevento, Italy; 4) Nephrology, Dialysis and Transplantation Unit, DETO, University of Bari “Aldo Moro”, Bari, Italy

**Keywords:** COVID-19, disinfection, ozone, Sars-CoV-2

## Abstract

Recent investigations have shown that severe acute respiratory syndrome coronavirus 2 (SARS-CoV-2) is able to resist on the surfaces and that the diffusion occurs through droplets that can remain suspended in the air as an aerosol.

The ozone generated *in situ* from oxygen is an active ingredient with a ‘biocidal’ action, but little is known about its capacity to inactivate specifically SARS-CoV-2.

Here we show, for the first time, the efficiency of the ozone treatment to neutralize the SARS-CoV-2 present in nasopharynx secretion samples with high viral load.

Our data show that ozone is effective in SARS-CoV-2 elimination.

## Highlights

• SARS-CoV-2 diffusion occurs through droplets that can remain suspended in the air as an aerosol and can deposit on surfaces.
• Here we show that ozone can inactivate the SARS-CoV-2 present in mucosal samples.
• Ozone treatment neutralizes SARS-CoV-2 open reading frame 1ab (ORF1ab) and the protein E and the nucleocapsid protein (N) genes.
• Ozone is effective for SARS-CoV-2 disinfection of work environments and living rooms.

## Introduction

In early January 2020, a novel Coronavirus was identified as an infective agent responsible for an outbreak of viral pneumonia in Wuhan, China [1].

SARS-CoV-2 is spread by human-to-human transmission: via respiratory droplets, through the saliva and aerosol secretions of the upper airways carried by cough and/or sneezing [2]; direct contact, with a handshake and touching the mucous membranes of the mouth, nose and eyes with contaminated hands; faecal-oral transmission [3].

While it is established that aerosol entry can take place not only through the airways but also through the conjunctival and oral mucous membranes, it is not clear whether a person can become infected even by touching surfaces or objects contaminated by the virus and then touching his mouth, nose and eyes.

Once expelled from the body, the virus, if contained in an aerosol, can remain suspended for a time that can vary depending on the size of the liquid or solid particles to which it may be attached. When deposited on different types of surfaces, it has a variable activity depending on the material it encounters, but the duration of its virulence is still under study [4].

For these reasons, it is important to establish a valid method to sanitize the workplace and the living rooms. Ozone generated *in situ* from oxygen is an active ingredient with a ‘biocidal’ action as a disinfectant for surfaces and drinking water and for use in cooling towers of industrial plants. Ozone has a microbicidal efficacy also on viruses [[5], [6], [7], [8], [9], [10], [11], [12], [13], [14]].

Ozone (O_3_) is unstable because gas degrades rapidly on its stable status, diatomic oxygen (O_2_) with the formation of atoms of free oxygen or free radical. Atoms of free oxygen or radicals are highly reactive, and they oxidize almost all (included virus, bacteria, organic and inorganic compounds) in contacts, making ozone a potent disinfectant and oxidant.

The literature has shown that in some clinical trials, ozone therapy has been given to patients with COVID-19, and these trials showed that this therapy had given benefits on patients, as reducing tissue hypoxia, decreasing hypercoagulability, renal and heart protection, modulating immune function, improving phagocytic function and impairing viral replication [[15], [16], [17], [18]]. Studies showed that ozone exposure eliminated heat-inactivated SARS-CoV-2 in different Personal Protective Equipment (PPE) components under appropriate exposure times, ozone concentrations, and relative humidity conditions, decreasing the risk of contamination associated with personal protective equipment management and increasing its availability [19]However, little is known about ozone capacity to inactivate specifically SARS-CoV-2. Here we show that ozone can inactivate the SARS-CoV-2 present in secretions from the nasopharynx.

## Materials and methods

Nasopharyngeal swabs were collected in duplicate from the 25 subjects. We collected, with the same swab, both the secretions from the nasopharynx and from the posterior pharyngeal wall and tonsillar pillars.

The study was carried out in accordance with the Helsinki Declaration and the European Guidelines for Good Clinical Practice, and written consent was obtained from all subjects.

The presence or absence of SARS-CoV-2 was detected by the SARS-CoV-2 Real-Time kit (SARS-CoV-2 Real-Time AA1571/96, Nuclear Laser Medicine s.r.l., Settala, Milano, Italy) using the CFX, Biorad Real-Time PCR before and after treatment with ozone. Specificity and the sensitivity of the used Real-Time kit is 100% and the detection limit of SARS-CoV-2 Real Time is 3,28 cp/reaction. All the experiments were performed following the SARS-CoV-2 Real-Time kit manufacturer guidelines.

Primers targeting the open reading frame 1ab (ORF1ab), protein E and the nucleocapsid protein (N) were amplified and examined, as indicated in the detection kit. The endogenous Internal Control (RNase P) was co-extracted and co-amplified in order to monitor all the procedures. For each patient, two swabs were collected. For ozone treatment, one of the two swabs were placed in a tube under a laminar flow hood (Top Safe model, Euroclone S.p.A) and were exposed to ozone generated by the Bio3gen apparatus (Finlinea s.p.a., Gazzaniga, Bergamo, Italy) with a flow rate of 3.6 L/min and an ozone output of 400 mg/h for a total time of 4 minutes. SARS-CoV-2 RNA was extracted by the NLM AA1571/96 Swab SARS-CoV-2 RNA rapid extraction kit (Nuclear Laser Medicine s.r.l., Settala, Milano, Italy) from ozone-treated and from not treated swabs, following the manufacturer guidelines, dissolving them in a tube containing 300 ul of RNA extraction solution. Samples were analysed by the SARS-CoV-2 Real-Time detection kit, as previously described.

## Results and discussion

### Ozone can neutralize the SARS-CoV-2

**Fig. 1:**
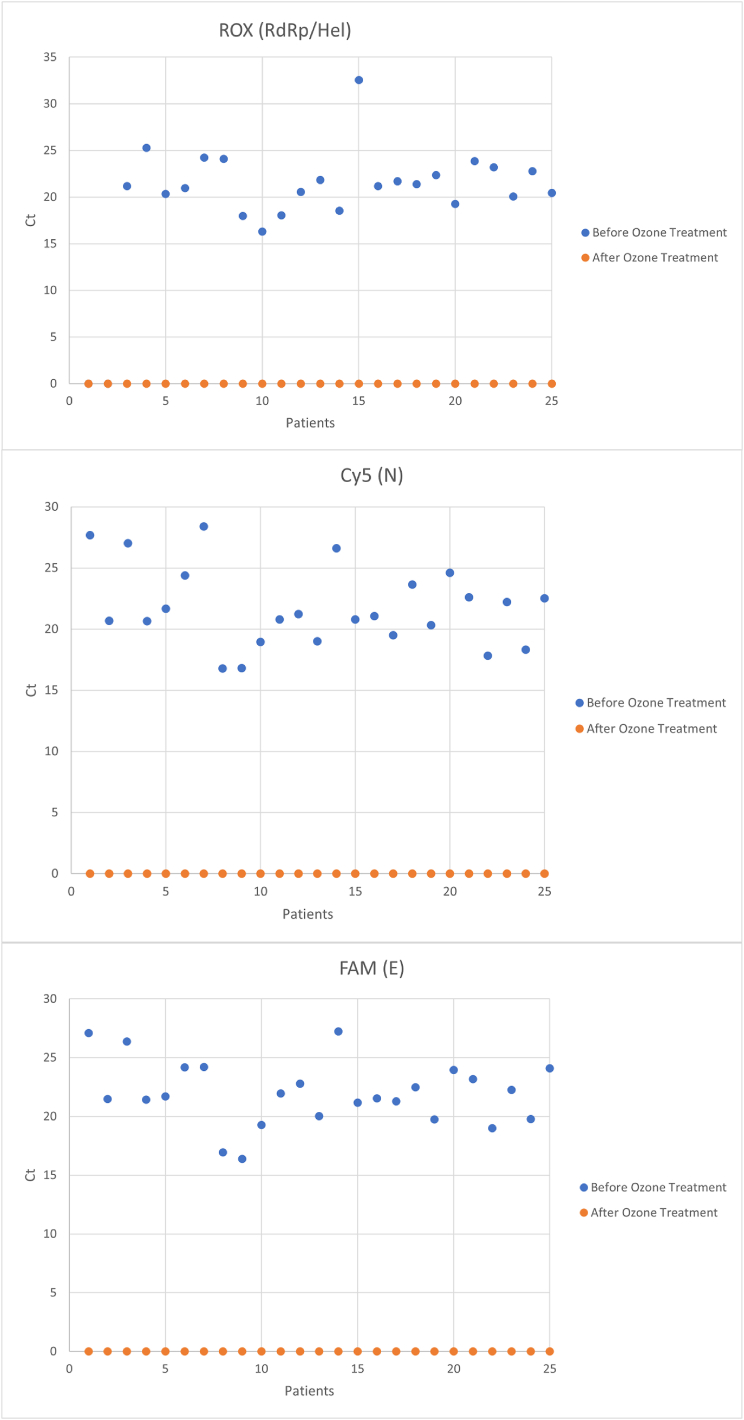
The graphs represent the Real-Time PCR results (Ct) of nasopharyngeal swab with samples before (colour blue) and after treatment (orange) with ozone for the three genes of SARS-CoV-2: ROX for RdRp and RdRp/Hel genus of the regions Orf1ab, Cy5 for N protein, FAM for E region. It is evident that, after ozone treatment, the viral regions are not amplificated.

The Real-time PCR on samples before the ozone treatment showed that our samples were all positive for the SARS-CoV-2 (Fig. 1 and Supplemental Table S1). Both the open reading frame 1ab (ORF1ab) and the protein E and the nucleocapsid protein (N) genes were positively amplified. Most of the samples had Ct values ranging from 16 and 22, showing that the viral load of our samples was very high (Fig. 1 Supplemental Table S1 and Supplemental Table S2).

Instead, after the ozone treatment, all 25 samples resulted negative for the virus, also samples with a high viral load, and no gene amplification for none of the three SARS-CoV-2 genes was detectable (Fig. 1 and Supplemental Table S3). These results showed, for the first time, the efficiency of an ozone treatment to neutralize the SARS-CoV-2. Its diffusion occurs through Flügge's micro-drops (droplets) and it is able to remain suspended in the air as an aerosol (core droplets). Flügge droplets that have a diameter >5 microns can spread up to 1m away [20]. The nuclei of the Flügge droplets (droplets nuclei) that have a diameter < 5 microns determine an aerosol that has a diffusion capacity of greater than 1 m [20].

Ozone is able to degrade rapidly organic compounds; at higher concentrations, ozone quickly inactivates a wide range of pathogens (bacteria, including spores, virus, protozoa) [5,[9], [10], [11]].

Referring specifically to effectiveness against SARS-CoV-2, considering the mechanism of action of ozone, a disinfectant action is conceivable. Nevertheless, at the moment, there are no direct demonstrations of efficiency obtained in controlled studies. This is one of the first studies that showed the effectiveness of ozone. Indeed, ozone is a stronger oxidant than the other common disinfectants, as chlorine and hypochlorite [21]. The use of chlorine or hypochlorite in many states has been reduced for the possibility of the formation of cancerogenic products as trihalomethanes during disinfection. On the contrary, disinfection with ozone does not produce detrimental residues and all residues of ozone will be converted into oxygen in a short time [21]. Ozone is considered an ecological disinfectant [17].

Moreover, ozone generators are currently promoted as dispositive usable for disinfection of work environments and living rooms. However, the operating conditions must be carefully selected since the effectiveness of the ozonation processes varies significantly depending on the characteristics of the environment to be sanitized [22].

Anyway, before the use of ozone in treatment locations, it is necessary to value the risk of exposition as for the operator engaged for disinfection operation, as for the staff that uses the disinfected place. Operators should be trained and provided with personal protection equipment [22].

In conclusion, here we showed that the ozone could inactivate the SARS-CoV-2 present in secretions from the nasopharynx, neutralizing the ORF1ab, the protein E and the N protein genes and our data support the effectiveness of SARS-CoV-2 ozone disinfection in work environments and living rooms. However, the ozone may have an effect on SARS-CoV-2 also *in vivo*, and therefore, further experiments will be needed to study *in vitro* and *in vivo* the ozone effect on the COVID-19. Moreover, since ground-level ozone concentrations peaked in summer [23,24], it is conceivable that ozone may contribute to the low levels of SARS-CoV-2 circulating in the warmer months.

## Transparency declaration

The authors declare no conflicts of interest.
