# Supplementary material for: Ozone eliminates novel coronavirus Sars-CoV-2 in mucosal samples
Source: New Microbes New Infect. 2021 Jul 24;43:100927. doi: 10.1016/j.nmni.2021.100927 (PMC8302481; doi:10.1016/j.nmni.2021.100927)
Supplement: Multimedia component 1 [file mmc1.pdf]

Ozone eliminates Novel Coronavirus Sars-CoV-2 in mucosal samples

SUPPLEMENTAL MATERIALS

Figure S1. Curves of Real Time relative fluorescence units of a representative sample (Patient ID 100900263) before (A) and after (B) the ozone treatment. In the graphs, each colour shows different donor fluorophores for the probes: Orange for ROX, which is a probe for RNA-dependent RNA polymerase (RdRp) and Rd/Rp helicase (Hel) of the region Open Reading Frame 1ab (Orf1ab); Green for Cy5, which indicates nucleocapsid (N) protein; Violet for fluorophore FAM for envelope (E) protein; Blue for JOE/HEX for the internal controls. For each gene/fluorophore, the PCR was carried out in the presence of sample and the internal positive control. Panel A shows positive samples before treatment with ozone, with amplification of both sample and internal controls for each gene/fluorophore. Panel B shows sample after treatment with ozone: only the internal control is amplificated, instead, the viral regions are not amplificated.

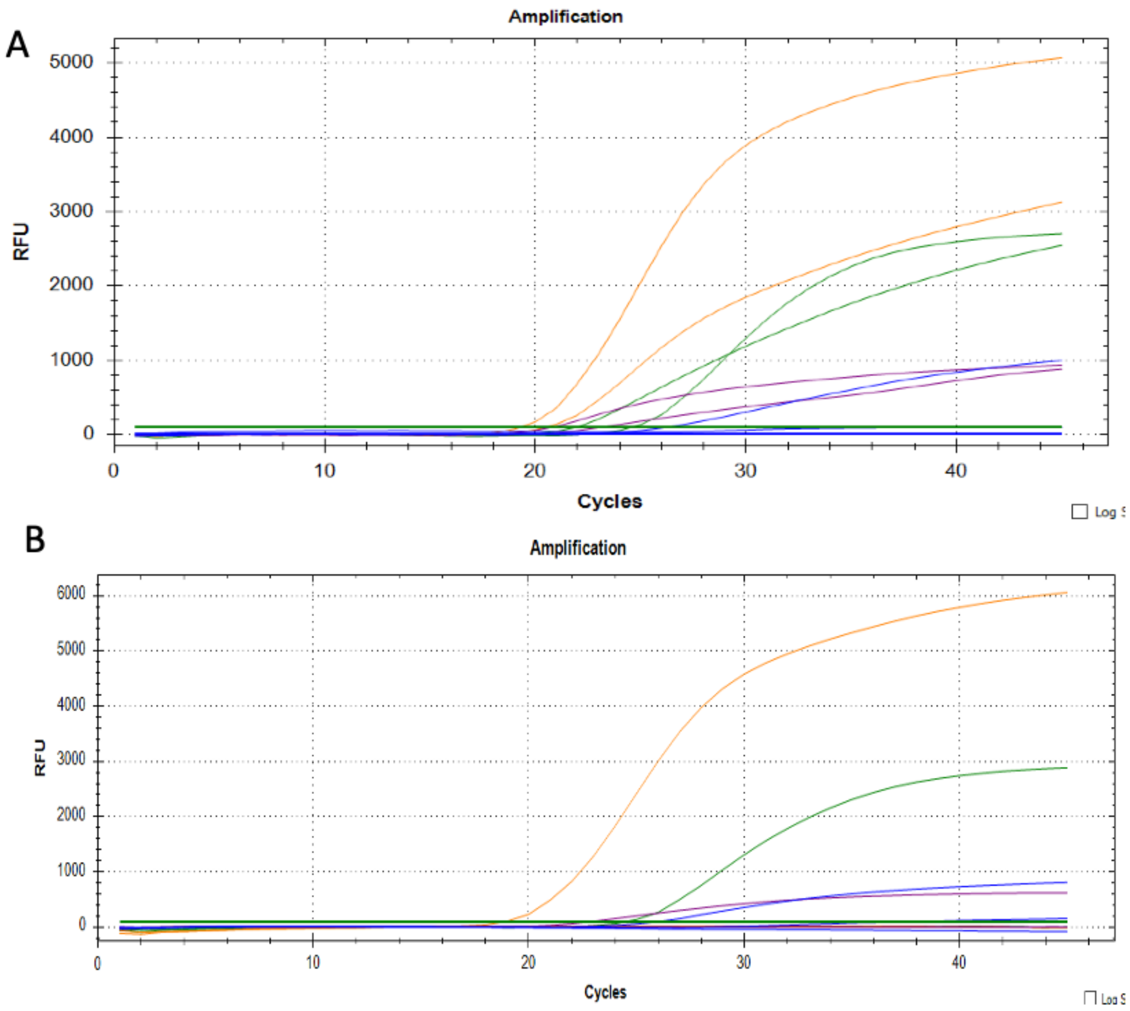

Table S1. Real time Ct values for the open reading frame 1ab (ORF1ab) the protein E and the nucleocapsid protein (N) genes of the SARS-CoV-2 before the ozone treatment. The PCR was carried out in the presence of two specific probes respectively for the internal control and for the viral regions. The donor fluorophore is different for the probes: FAM for E region, ROX for RdRp and RdRp/Hel genus of the regions Orf1ab, Cy5 for N and JOE/HEX for the internal control.

**Patient ID**	**ROX (RdRp/Hel) Ct**	**RdRp/Hel positive control Ct**	**Cy5 (N) Ct**	**N positive control Ct**	**FAM (E) Ct**	**E positive control Ct**	**Results**
100700152	N/A	19,73	27,68	23,08	27,09	24,12	SARS-CoV-2 Positive
100700151	21,19		20,68		21,47		SARS-CoV-2 Positive
100700184	25,27	19,20	27,02	22,84	26,37	24,50	SARS-CoV-2 Positive
100700170	20,35		20,64		21,41		SARS-CoV-2 Positive
100700157	20,97		21,67		21,70		SARS-CoV-2 Positive

<table>
<tr><td>100900097</td><td>24,25</td><td rowspan="4">19,41</td><td>24,38</td><td rowspan="4">23,18</td><td>24,17</td><td rowspan="4">23,65</td><td>SARS-CoV-2 Positive</td></tr>
<tr><td>100900060</td><td>24,1</td><td>28,39</td><td>24,2</td><td>SARS-CoV-2 Positive</td></tr>
<tr><td>100900102</td><td>17,96</td><td>16,78</td><td>16,94</td><td>SARS-CoV-2 Positive</td></tr>
<tr><td>100900101</td><td>16,31</td><td>16,81</td><td>16,40</td><td>SARS-CoV-2 Positive</td></tr>
<tr><td>100900123</td><td>18,04</td><td rowspan="4">19,32</td><td>18,96</td><td rowspan="4">23,08</td><td>19,27</td><td rowspan="4">24,56</td><td>SARS-CoV-2 Positive</td></tr>
<tr><td>100900263</td><td>20,54</td><td>20,78</td><td>21,95</td><td>SARS-CoV-2 Positive</td></tr>
<tr><td>100900150</td><td>21,82</td><td>21,23</td><td>22,77</td><td>SARS-CoV-2 Positive</td></tr>
<tr><td>100900187</td><td>18,53</td><td>19,01</td><td>20,02</td><td>SARS-CoV-2 Positive</td></tr>
</table>

100900330	32,55	19,70	26,60	23,33	27,22	23,88	SARS-CoV-2 Positive
100900322	21,19		20,80		21,16		SARS-CoV-2 Positive
100800032	21,69	19,87	21,07	25,05	21,52	24,12	SARS-CoV-2 Positive
100800056	21,40		19,51		21,29		SARS-CoV-2 Positive
101000018	22,35	19,68	23,64	23,09	22,48	23,68	SARS-CoV-2 Positive
101000020	19,25		20,31		19,75		SARS-CoV-2 Positive
101200081	23,86	19,49	24,6	23,04	23,96	23,56	SARS-CoV-2 Positive
101200038	23,18		22,61		23,17		SARS-CoV-2 Positive

<table>
<tr><td>100700010</td><td>20,06</td><td>20,06</td><td>17,81</td><td>23,01</td><td>19,01</td><td>23,91</td><td>SARS-CoV-2 Positive</td></tr>
<tr><td>100800215</td><td>22,78</td><td>19,47</td><td>22,21</td><td>23,23</td><td>22,25</td><td>23,68</td><td>SARS-CoV-2 Positive</td></tr>
<tr><td>100800098</td><td>20,43</td><td rowspan="2">20,44</td><td>18,33</td><td rowspan="2">23,83</td><td>19,78</td><td rowspan="2">24,61</td><td>SARS-CoV-2 Positive</td></tr>
<tr><td>101100056</td><td>21,23</td><td>22,51</td><td>24,1</td><td>SARS-CoV-2 Positive</td></tr>
</table>

Table S2. Correspondence between viral copy number and Ct for RdRp and RdRp/Hel gene.

SARS-CoV-2 (Log_{10}) cp/ml	SARS-CoV-2 Ct RdRp and RdRp/Hel gene Ct (ROX)
2,0	33-36
3,0	30-33
4,0	27-30
5,0	24-27
6,0	21-24
7,0	17-21
8,0	14-17

Table S3. Real time Ct values for the open reading frame 1ab (ORF1ab) the protein E and the nucleocapsid protein (N) genes of the SARS-CoV-2 after the ozone treatment. The PCR was carried out in the presence of two specific probes respectively for the internal control and for the viral regions. The donor fluorophore is different for the probes: FAM for E region, ROX for RdRp and RdRp/Hel gene of the regions Orf1ab, Cy5 for N and JOE/HEX for the internal control.

Patient ID	ROX (RdRp/Hel) Ct	RdRp/Hel positive control Ct	Cy5 (N) Ct	N positive control Ct	FAM (E) Ct	E positive control Ct	Results
100700152	N/A	19,47	N/A	23,23	N/A	23,68	SARS-CoV-2 Negative
100700010	N/A		N/A		N/A		SARS-CoV-2 Negative
100700184	N/A		N/A		N/A		SARS-CoV-2 Negative
100700151	N/A		N/A		N/A		SARS-CoV-2 Negative
100700170	N/A		N/A		N/A		SARS-CoV-2 Negative
100700157	N/A		N/A		N/A		SARS-CoV-2 Negative
100800032	N/A		N/A		N/A		SARS-CoV-2 Negative
100800098	N/A		N/A		N/A		SARS-CoV-2 Negative
100800056	N/A		N/A		N/A		SARS-CoV-2 Negative
100800215	N/A		N/A		N/A		SARS-CoV-2 Negative
100900123	N/A	18,92	N/A	22,92	N/A	24,54	SARS-CoV-2 Negative
100900097	N/A		N/A		N/A		SARS-CoV-2 Negative
100900263	N/A		N/A		N/A		SARS-CoV-2 Negative
100900060	N/A		N/A		N/A		SARS-CoV-2 Negative
100900102	N/A		N/A		N/A		SARS-CoV-2 Negative
100900150	N/A		N/A		N/A		SARS-CoV-2 Negative

100900101	N/A		N/A		N/A		SARS-CoV-2 Negative
100900187	N/A		N/A		N/A		SARS-CoV-2 Negative
100900330	N/A		N/A		N/A		SARS-CoV-2 Negative
100900322	N/A		N/A		N/A		SARS-CoV-2 Negative
101000018	N/A	19,70	N/A	23,33	N/A	25,49	SARS-CoV-2 Negative
101000020	N/A		N/A		N/A		SARS-CoV-Negative
101100056	N/A	19,29	N/A	23,36	N/A	23,84	SARS-CoV-2 Negative
101200081	N/A		N/A		N/A		SARS-CoV-2 Negative
101200038	N/A		N/A		N/A		SARS-CoV-2 Negative
